# Understanding Factors Associated with Motivation to Quit Vaping among Vapers in the Federal Territory of Kuala Lumpur, Malaysia

**DOI:** 10.3390/healthcare11141980

**Published:** 2023-07-08

**Authors:** Lei Hum Wee, Jo Ann Andoy Galvan, Sapna Shridhar Patil, Priya Madhavan, Dinesh Mahalingam, Chai Hong Yeong, Yin How Wong, Hui Qi Poh, Sanjay Suthahar, Shamella Diya David, Xiao Jing Tan, Izzah Athirah Rosli, Caryn Mei Hsien Chan, Nizam Baharom, Nor Asiah Muhamad, Ching Sin Siau

**Affiliations:** 1 Centre for Community Health Studies, Faculty of Health Sciences, Universiti Kebangsaan Malaysia, Kuala Lumpur 50300, Malaysia; weeleihum@ukm.edu.my (L.H.W.); caryn@ukm.edu.my (C.M.H.C.); 2 School of Medicine, Faculty of Health & Medical Sciences, Taylor’s University, 1 Jalan Taylors, Subang Jaya 47500, Malaysia; joannandoy.galvan@taylors.edu.my (J.A.A.G.); sapnashridhar.patil@taylors.edu.my (S.S.P.); priya.madhavan@taylors.edu.my (P.M.); dinesh.m@taylors.edu.my (D.M.); chaihong.yeong@taylors.edu.my (C.H.Y.); yinhow.wong@taylors.edu.my (Y.H.W.); pohhuiqi@sd.taylors.edu.my (H.Q.P.); sanjaysuthahar@sd.taylors.edu.my (S.S.); shamelladiyadavid@sd.taylors.edu.my (S.D.D.); xiaojing.tan@sd.taylors.edu.my (X.J.T.); 3 Non-communicable Diseases and Public Health Research Group, School of Medicine, Faculty of Health & Medical Sciences, Taylor’s University, 1 Jalan Taylors, Subang Jaya 47500, Malaysia; 4 Medical Advancement for Better Quality of Life Impact Lab, Taylor’s University, 1 Jalan Taylors, Subang Jaya 47500, Malaysia; 5 SP Care Group, Level 3, Menara SP Care, Rawang Sentral, Rawang 48000, Malaysia; 6 Sector for Evidence-Based Healthcare, National Institutes of Health, Ministry of Health, Shah Alam 40170, Malaysia; izzahrosli97@gmail.com (I.A.R.); drnorasiah@moh.gov.my (N.A.M.); 7 Faculty of Medicine and Health Sciences, Universiti Sains Islam Malaysia, Nilai 71800, Malaysia; drnizamb@usim.edu.my

**Keywords:** e-cigarette, vape, nicotine dependence, quit attempts, motivation, Malaysia

## Abstract

The prevalence of vaping worldwide is showing an upward trend. This study aimed to determine the factors associated with motivation to quit vaping among vapers in the Federal Territory of Kuala Lumpur, Malaysia, through a cross-sectional, purposive sampling study. Respondents were required to complete a questionnaire consisting of vapers’ sociodemographic questions, habitual behavioral pattern questions, the e-Fagerström Test of Nicotine Dependence, the Glover–Nilsson Smoking Behavioral Dependence Questionnaire, perception questions, motivation to quit questions, and withdrawal symptom questions. A total of 311 vapers participated in this study. The majority of the vapers were male (84.6%), younger (18–25 years) (55.3%), and with monthly income less than RM 4000 (USD 868; 83.9%). The level of motivation to quit vaping was found to have a significant association with the perception of vaping being as satisfying as cigarette smoking (*p* = 0.006) and mild to very strong nicotine dependence (*p* = 0.001). Participants who recorded moderate and strong habitual vaping behaviors had lower odds of having high motivation to quit vaping compared to those recording slight habitual behaviors (OR = 0.279, 95%CI(0.110–0.708), *p* = 0.007 and OR = 0.185, 95%CI(0.052–0.654), *p* = 0.009, respectively). Factors associated with higher motivation to quit vaping could be explored to gain better understanding of how to increase their motivation level for future quit attempts.

## 1. Introduction

Vapes, also known as e-cigarettes (ECs), employ an “e-liquid” that typically includes flavorings, propylene glycol, vegetable glycerin, nicotine produced from tobacco, and other substances. An aerosol is then produced by heating the liquid, which the user inhales. In 2016, the FDA classified vapes with nicotine as tobacco products because they can cause addiction [1].

Currently, there is a surge in the usage of vapes, and it has been found that vapes are used as an alternative to smoking conventional cigarettes due to the belief that they cause less harm and have fewer toxins, despite their addictiveness [2,3]. Tehrani et al. [4] reported that vaping has a 23% lifetime prevalence and an 11% current prevalence worldwide. According to Driezen et al. [5], 33.7% of Malaysians stated that they had never used an EC, 2.3% had done so monthly, 3.7% had done so on a weekly basis, and 5.4% had done so on a daily basis. In 2019, the National Health and Morbidity Survey Malaysia also showed that the percentage of EC use in the previous month among young adults was higher than that of older adults: 7.5% (15–19 years old), 14.7% (20–24 years old), and less than 5% (≥30 years old) [6]. However, as vapers continue to vape, research in Malaysia and elsewhere show that they have higher chances of vaping exclusively [3,7]. In 2018, approximately 4 in 10 Malaysian students were exclusive EC users [8].

Several studies have attempted to chart the characteristics of EC users. A study conducted among Malaysian young adults revealed that the mean age of initiation of vaping was 26.8 years old and most of them vaped every day (88.3%). Moreover, the majority of them were non-dual users (80.3%), with 78.3% reporting having less than 20 vaping sessions daily and a mean number of puffs per session of 19.24 [9]. The main reasons that the vapers started vaping was to quit tobacco cigarettes (62.4%), and most of them had low motivation to stop vaping (92.6%) [9]. Another local study found that the mean age of vapers was 33 years old, and most of them were non-dual users (99.4%) [3]. Additionally, Ling et al.’s [10] study conducted among 22,228 Malaysian respondents aged 13–18 years showed that ethnicity was significantly associated with EC use, in which the Malay ethnic group had the highest proportion of EC use compared to the other ethnic groups, at 76.6%. Moreover, according to a study by Afolalu et al. [11] conducted in Japan, manual workers were more likely to use ECs (45%). A study by Cox et al. [12] demonstrated that utilizing e-liquids that have a greater nicotine strength may be advised if compensatory puffing practices are associated with using e-liquids with a lower nicotine strength that results in higher toxicant exposure.

Despite its increasing prevalence, studies have shown that among current users of ECs, some have reported a previous attempt at quitting vaping, or have the intention to quit vaping in the future [13,14,15]. Palmer et al. [14], in a study amongst adult EC users, found that 15.2% reported a past-year quit attempt and 60.7% reported planning to quit vaping in the future. Another study using 2015–2016 data in the US showed that 62.4% intended to quit vaping for good, whilst 25% had a quit attempt in the prior year [15]. A study in Central and Eastern Europe found that the prevalence of ever quitting vaping was 13.9%, whilst 25.2% reported an intention to quit vaping in the future [13].

A number of factors are involved in the motivation to quit vaping. In a study conducted by Kale et al. [16], one third of current vapers were motivated to quit vaping due to COVID-19-related issues. Another study conducted by Smith et al. [17] revealed that among those who had thoughts of quitting vape, nearly half were male (46.15%), had annual household incomes above 50,000 USD (43.18%), had a frequency of vaping in the past 30 days of less than four times (47.42%), and had anxiety (44.18%) and depression (46.81%) symptoms in the past year. A systematic review on motivation to quit vaping found that adults were more motivated to quit vaping due to its cost, lower satisfaction from EC use, and psychological concerns [18].

In the Asian context, many studies have focused on EC initiation and ever or current use (e.g., Hairi et al. and Patanavanich et al. [19,20]), whilst studies on the motivation and intention to quit vaping are scarce. A study among 429 EC users showed that 65.3% reported an intention to quit vaping, and this intention was related to earning a lower income and perceiving that smoking ECs was more expensive compared to smoking cigarettes [21]. Based on the literature reviewed above, it is evident that there is a lack of studies in Malaysia targeted at investigating the motivation level to quit vaping and its related factors. Such studies are direly needed to build evidence-based interventions that can help individuals increase their motivation to quit vaping, thus increasing their likelihood of vaping cessation. This study aimed to determine the factors associated with motivation to quit vaping among young adults in Malaysia. We hypothesized that those with higher nicotine dependence, positive perceptions toward vaping, and higher withdrawal symptoms would record lower motivation to quit vaping.

## 2. Materials and Methods

### 2.1. Study Design, Setting and Participants

This research was conducted using a cross-sectional study design, and data were collected in February 2023. Respondents were vapers recruited from shopping complexes and restaurants in the town of Rawang, Gombak District, Federal Territory, Kuala Lumpur, Malaysia.

The study’s inclusion criteria were Malaysian individuals who vaped with nicotine-containing e-liquids for the past month and who were at least 18 years old. Those who refused to participate in the study, solely smoked conventional cigarettes, or had stopped vaping were excluded from the study. A sample size calculation was performed using the Open Epi website, indicating a minimum of 292 respondents required for this study. A small token was given to all respondents who completed the questionnaires.

### 2.2. Study Instruments

A structured, closed-ended, and validated questionnaire in Bahasa, Malaysia, was used in this study. The questionnaire used had the following sections: (1) Sociodemographic background information, such as gender, age, ethnicity, marital status, education level, working status, stress, monthly income, health problems (if any), and health status, (11 questions). (2) EC users’ profiles, including EC use, age of EC initiation, daily/non-daily use of ECs, and main reasons for using ECs (4 questions). (3) Habitual vaping behavioral pattern, including number of EC sessions and puffs, common brands of EC liquid used, flavor, money spent on purchasing e-liquids, volume of e-liquids (mL), concentration of e-liquid used (mg/mL), common places of EC use, difficulties in restriction to use ECs in non-smoking areas, location of e-liquid purchases, and experiences of health effects associated with EC use (11 questions). (4) e-Fagerström Test of Nicotine Dependence (6 questions) [22], with scores of 1–3 = low dependence, 4–6 = moderate dependence, and 7–10 = high dependence. (5) Glover–Nilsson Smoking Behavioral Dependence Questionnaire regarding vaping (11 questions), with scores of 1–6 = light, 7–11 = mild, 12–22 = moderate, 23–33 = strong, and >33 = very strong [23]. (6) Perception of benefits, harm, and risks, assessed using a 10-item yes/no questionnaire comparing EC use with conventional cigarettes, such as ECs providing the throat hit sensation, equal satisfaction, ECs being like conventional cigarettes and being a safer alternative, reducing the use of conventional cigarettes, leading to no urges to smoke conventional cigarettes or urges to use ECs being at the same level as conventional cigarettes, always using ECs in non-smoking areas, ECs helping to quit conventional cigarettes, and being able to use a higher amount of nicotine in ECs [22]. (7) Quitting vaping, measured using questions about having ever tried to stop vaping, having any successful attempt to quit vaping, methods used to quit vaping, and plans regarding quitting vaping in the last 12 months (4 questions). (8) Motivation to quit vaping was measured using a single-item question, with scores of 1–5 = low motivation and 7- 10 = high motivation (7 response options) [24]. (9) Withdrawal symptoms, with a higher total score indicating higher withdrawal symptoms (9 questions) [25]. (10) Dual users’ profiles: Smoking conventional cigarettes, daily or non-daily smokers, age of initiation, total number of cigarettes smoked daily, money spent on cigarettes, and comparison of satisfaction between smoking cigarettes and vaping and reasons why (10 questions). (11) Intention to quit (1 question). Details of the questions of the used questionnaires have been published elsewhere [9].

### 2.3. Data Collection

Vapers were approached by the researchers in shopping malls and restaurants. Those who were seen to have/hold an EC were approached and asked if they were interested in participating in the study. Vapers who agreed to participate were further asked if they used ECs with nicotine for the past month, and those who answered “yes” were recruited as study respondents. Printed self-administered questionnaires were used. The respondents provided informed consent prior to completing the questionnaire. The time taken to complete the questionnaires was approximately 10–12 min.

### 2.4. Statistical Analysis

Analysis was performed using IBM Statistical Package and Social Sciences (SPSS Inc. Chicago, IL, USA), version 28.0. Descriptive statistics were used to determine the sociodemographic characteristics and vaping profiles of the EC users. Chi-square tests were conducted to determine the association between the factors associated with the level of motivation to quit vaping. Any significant associations were further analyzed using multiple logistic regression to examine the relationship between the associated factors and the level of motivation to quit vaping. Continuous variables are described as means and standard deviations (SDs). All reported *p*-values are two-tailed, and *p* < 0.05 was considered statistically significant.

## 3. Results

Table 1 shows the sociodemographic characteristics and vaping profiles among vapers in the Federal Territory of Kuala Lumpur, Malaysia. A total of 311 respondents were identified. Most of the respondents were male (84.6%), aged 18–25 years old (55.3%) with a mean age of 27.4 years (SD 8.2), single (67.2%), had completed tertiary education (55.3%), and had a monthly income of RM 4000 or less (USD 902.73) (83.9%). Approximately half of the vapers were either stressed (39.2%) or very stressed (10.0%). The majority of the respondents recorded no health problems (90.7%) and rated their health as “good” (63.3%) or “very good” (20.3%) (Table 1).

More than half of the respondents started vaping at the age of 19 years or younger (51.8%), and 37.6% of them began vaping between the ages of 20 and 29 and vaped daily (68.2%). The three main reasons for starting to vape were “to try” (29.3%), “to stop smoking cigarettes or tobacco products” (28.3%), and “to replace cigarettes or tobacco products” (20.6%). Additionally, the majority of the vapers preferred fruit-flavored e-liquids (53.1%). The mean daily usage of ECs was 23.95 sessions, while the average number of puffs per session was 26.42. Most of the vapers remained “unsure” or “had no intention” about their decision to stop vaping within the next three months (64.6%). Approximately 67% of the vapers were sole vapers (66.8%) and those who combined vaping with smoking conventional cigarettes recorded a daily mean of 3.02 sticks (Table 1).

### Factors Associated with Motivation to Quit Vaping

Table 2 shows the association between factors that promote the level of motivation to quit vaping. The level of motivation to quit vaping was found to have a significant association with the perception of vaping being as satisfying as smoking cigarettes (*p* = 0.006). There was a significant association between those who ranked their nicotine dependency as mild to very strong (*p* = 0.001). There was also a significant difference among respondents experiencing withdrawal symptoms: Feeling depressed (*p* = 0.004), frustrated and angry (*p* = 0.017), fearful and worried (*p* = 0.009), finding it difficult to focus (*p* = 0.022), feeling anxious (*p* = 0.012), and having an increased appetite (*p* = 0.010) (Table 2).

Further analysis using binary logistic regression was conducted to observe the relationship between sociodemographic characteristics, vaping profile, daily amount of vaping, and habitual vaping behavior and a high level of motivation to quit vaping (Table 3). Based on the results, participants with an income of RM 1000–1999 (OR = 0.273, 95% CI = 0.094–0.788, *p* = 0.016), RM 2000–2999 (OR = 0.237, 95% = CI 0.088–0.639, *p* = 0.004), RM 4000–4999 (OR = 0.116, 95% CI = 0.015–0.915, *p* = 0.041), and RM 5000 and above (OR = 0.126, 95% CI = 0.026–0.998, *p* = 0.050) were less likely to have high motivation to quit vaping in comparison to those who earned less than RM 1000. With regard to the daily number of vaping sessions, participants with 11–20 sessions (OR = 9.917, 95% CI = 1.255–78.376, *p* = 0.030) or more than 30 sessions of vaping per day (OR = 10.928, 95% CI = 1.211–98.612, *p* = 0.033) had higher odds of having higher motivation to quit vaping compared to those who had 10 or less sessions per day. Concerning behavioral dependence related to vaping, participants who recorded moderate and strong habitual vaping behavior were less likely to have high motivation to quit vaping compared to those with slight habitual behavior (OR = 0.279, 95% CI = 0.110–0.708, *p* = 0.007 and OR = 0.185, 95% CI = 0.052–0.654, *p* = 0.009, respectively). Those who perceived vaping as being as satisfying as smoking tobacco cigarettes were also less likely to have high motivation to quit vaping (OR = 0.377, 95% CI = 0.186–0.799, *p* = 0.007).

## 4. Discussion

Low motivation to quit, positive perception toward vaping, and moderate and strong habitual vaping behavior may indicate future long-term, with vaping potentially mimicking conventional cigarette smoking [26]. Based on characteristics, profile, behavior addiction, and motivation, this study indicated that vapers are unlikely to make quit attempts and to successfully regulate the cravings, physical withdrawal, and negative affect that accompany quit attempts. It has been reported by Hopkins et al. that the majority of smokers experience fluctuations in their motivation to quit, with daily dynamic changes predicting relapse [27]. Our study reported similar findings for smokers, with those who had more vaping sessions per day (>10 sessions) also showing higher odds of having high motivation to quit vaping. In addition, vapers with higher income had lower motivation to quit vaping, which is consistent with several other studies [8,24]. However, our results were not consistent with a study conducted among English adults, where higher income was associated with higher motivation to quit vaping, but this significant association became non-significant after controlling for education level [28]. This may be because individuals from a higher SES background have greater exposure to EC advertisements. Groups with a SES may be targeted, as they have been found to be early supporters of novel technologies [29] or to have extra money to spend on new products [30].

When vapers compared their satisfaction from vaping with their satisfaction from tobacco cigarettes, those who found vaping to be as satisfying were less motivated to quit vaping. It is known that one of the factors that encourage users to switch to other products beside cost and accessibility is when the new product provides a similar—if not higher—satisfactory level [31]. This is supported by a study conducted by Gravely et al. [32], who found that lack of satisfaction with vaping leads to stopping regular EC usage. This study concluded that the trend in tobacco consumption is shifting from conventional cigarettes to exclusive vaping, especially among the younger age group. There is a rapid increase in EC usage, especially among those who were tobacco cigarettes users [28]. Additionally, this study showed that the majority are sole vapers (66.8%), which contradicts the study conducted locally by Wan Puteh et al., which revealed that the majority were dual users (40.3%) [8]. This could indicate the current vaping trend in Malaysia, which has seen a shift from dual users to sole users, especially among the younger age group.

It is interesting to note that those who engaged in more vaping sessions had higher odds of being motivated to make quit attempts compared to those with fewer daily vaping sessions. This could be because users experience too many sessions, coupled with the need to recall many puffs for each session. This reinforces the need for researchers around the world to identify ways to accurately measure vapers’ nicotine and behavior dependence in relation to vaping compared to conventional cigarettes, rather than depending solely on cotinine tests, which can be costly and time consuming in clinical practice. Farsalinos et al. argued that relying only on sessions and puffs may not be an appropriate indicator of vaping behavior and dependency because individual characteristics, such as inhaling technique and lung capacity, might considerably alter nicotine absorption [32].

Failures in quitting nicotine use and experiencing withdrawal symptoms are associated with heavier nicotine dependence [33]. It was also found in this study that respondents with high nicotine dependence and those who experience withdrawal symptoms were less likely to be motivated to quit [34,35]. This could potentially be explained by a study conducted by Horn et al. [36] on tobacco users, where experiencing more symptoms of dependence (withdrawal symptoms) was negatively associated with the ability to quit smoking.

It is widely reported that the initiation age of tobacco consumption is getting younger with the emergence of ECs [35]. This study showed that the initiation age of vaping is 19 years old or younger (51.8%), which is consistent with another study [37]. The main reasons for initiation were “to try” (29.3%), “to stop smoking cigarettes or tobacco products” (28.3%), and “to replace cigarettes or tobacco products” (20.6%); this contradicts a study conducted by Lewek et al. that reported that the main reasons include “lack of unpleasant odor” (65.7%), “e-cigarettes are less harmful than cigarettes” (64.6%), and “better taste” (58.8%) [38]. The most preferred flavor e-liquid was fruit (53.1%), and this is consistent with local and international studies [8,39].

This study has a few strengths and limitations. First, it intended to control the characteristics of respondents from the same district and used printed questionnaires to limit the distribution so as to overcome the weaknesses of online surveys. It is also hoped that the printed questionnaires increased the respondent’s perception that their responses had greater anonymity, hence encouraging them to respond more honestly. The questionnaire was explicitly designed to be completed by respondents without interviewer bias during the process. In terms of study limitations, this was a cross-sectional study that used purposive sampling; hence, we are not able to make causal inferences, and the study may have been susceptible to sampling bias. Data from the returned questionnaires may not represent the population. Additionally, the number of respondents who declined to participate in the study was not recorded, meaning that we are not able to provide a non-response rate. Future studies should consider comparing vaping characteristics and intention to quit vaping stratified by gender and other demographic factors.

## 5. Conclusions

The group shared some similarities and differences in their demographics and vaping profiles. The range of patterns used and preferences in vaping indicate more research is needed to identify what is the best specific measure to explain the behavioral pattern associated with vaping and addiction to nicotine so that we can confidently understand how to increase vapers’ motivation level for future quit attempts. The issues with the initiation, persistence, escalation, and positive perception toward vaping and not thinking about quitting constitute a real public health issue that is not to be taken lightly.

## Figures and Tables

**Table 1:** Sociodemographic characteristics and vaping profiles of the respondents (*N* = 311).

Sociodemographic Characteristics	Frequency (*n*)	Percentage (%)
Gender		
Male	263	84.6
Female	48	15.4
Age (in years)		
Mean (SD), 27.4 (8.2)		
18–25	172	55.3
26–35	98	31.5
36–45	31	10.0
46–55	6	1.9
56 and above	4	1.3
Race		
Malay	179	57.6
Chinese	82	26.4
Indian	41	13.2
Other	9	2.1
Marital status		
Single	209	67.2
Married	92	29.6
Divorced/widowed	10	3.2
Education level		
Primary	8	2.6
Secondary	131	42.1
Tertiary	172	55.3
Work status		
Government	25	8.0
Private	119	38.3
Self-employed	80	25.7
Student	73	23.5
Retired or unemployed	14	4.5
Stress level		
No stress	65	20.9
Somewhat stressed	93	29.9
Stressed	122	39.2
Very stressed	31	10.0
Monthly income * MYR (USD)		
<MYR 1000 (<225.30)	70	22.5
MYR 1000–1999 (225.30–450.38)	58	18.6
MYR 2000–2999 (450.60–675.68)	79	25.4
MYR 3000–3999 (675.90–900.98)	54	17.4
MYR 4000–4999 (901.21–1126.28)	26	8.4
≥MYR 5000 (≥1126.51)	24	7.7
Health problems		
No	282	90.7
Yes	29	9.3
Health level		
Not sure	18	5.8
Not good	3	1.0
Not so good	30	9.6
Good	197	63.3
Very good	63	20.3
Age of initiation (years)		
Mean (SD), 20.94 (7.80)		-
Below 10	4	1.3
10–19	157	50.5
20–29	117	37.6
30–39	19	6.1
40 and above	14	4.5
Daily consumption		
Everyday	212	68.2
Non-daily	99	31.8
Reasons for initiation		
To try	91	29.3
To stop smoking cigarettes/ tobacco products	88	28.3
To replace cigarettes/ tobacco products	64	20.6
To reduce the number of cigarettes/ tobacco products used	39	12.5
To vape in a non-smoking area	14	4.5
To reduce the cost of smoking	9	2.9
Other	6	1.9
Average daily usage of ECs (session)		
Mean (SD), 23.95 (41.62)		-
10 or less	153	49.2
11–20	75	24.1
21–30	30	9.6
31 and above	53	17.0
Puffs per session		
Mean (SD) 23.73 (46.51)		-
1–25	260	83.6
26–50	24	7.7
51–75	2	0.6
76–100	10	3.2
>101	15	4.8
Flavors		
Fruit	165	53.1
Mint	64	20.6
Tobacco	36	11.6
Coffee/tea	28	9.0
Other (beans, desserts, sweets, etc.)	18	5.7
Intention to stop vaping within next 3 months		
Not sure	126	40.5
Yes	110	35.4
No	75	24.1
Use of other tobacco products		
Sole vaper	208	66.8
Dual user (ECs and cigarettes)	103	33.1
Average daily tobacco cigarettes smoked		
Mean (SD), 3.02 (5.65)		-
1–5	49	15.8
6–10	52	16.7
11–15	10	3.2
16–20	28	9.0
21 and above	11	3.5

* MYR 1 = USD 0.22 as of 29 May 2023. EC, e-cigarettes.

**Table 2:** Association between factors that promote the level of motivation to quit vaping (*N* = 311).

	Motivation to Quit Vaping		χ^2^	*p*-Value ^a^
	Low *n* (%)	High *n* (%)		
Perception of the benefits, harm, and risks of vaping				
I get a definite nicotine hit from ECs	150 (88.8)	19 (11.2)	0.568	0.451
Vaping is as satisfying as tobacco smoking	155 (92.3)	13 (7.7)	7.682	0.006 *
I like ECs because they look and feel like a cigarette	99 (86.8)	15 (13.2)	0.063	0.802
Vaping feels healthier than smoking	171 (87.2)	25 (12.8)	0.022	0.881
Vaping has helped me to cut down on tobacco smoking	203 (88.3)	27 (11.7)	0.517	0.472
I don’t have the urge to smoke as much since using ECs	199 (89.6)	23 (10.4)	3.361	0.067
I crave ECs as much as I do/did tobacco	119 (88.8)	15 (11.2)	0.389	0.533
I frequently use ECs in places where tobacco smoking is banned	115 (89.8)	13 (10.2)	1.127	0.288
ECs have helped me to stop smoking	186 (87.7)	26 (12.3)	0.046	0.830
ECs allow me to use nicotine more	121 (87.7)	17 (12.3)	0.011	0.916
Level of nicotine dependence (Fagerström Test for Nicotine Dependence)			2.539	0.281
Low dependence (1–3)	115 (84.6)	21 (15.4)		
Moderate dependence (4–6)	86 (87.8)	12 (12.2)		
High dependence (7–10)	70 (92.1)	6 (7.9)		
Vaping after waking up (1st EC of the day)			1.713	
Within 5 min	84 (90.3)	9 (9.7)		0.634
6–30 min	55 (88.7)	7 (11.3)		
31–60 min	47 (87.0)	7 (13.0)		
After 60 min	86 (84.3)	16 (15.7)		
Vaping behavior (Glover–Nilsson Smoking Behavioral Questionnaire) This scales’ scores indicate the nicotine behavioral dependency ranking			19.043	
Slight (1–6)	32 (69.6)	14 (30.4)		0.001 *
Mild (7–11)	40 (83.3)	8 (16.7)		
Moderate (12–22)	109 (90.8)	11 (9.3)		
Strong (23–33)	66 (94.4)	4 (5.7)		
Very strong (34–44)	25 (92.6)	2 (7.4)		
Withdrawal symptoms (Minnesota Nicotine Withdrawal Syndrome) Have you ever experienced the following symptoms during the use of e-cigarettes? The total score of the scale ranges from 0 to 36, depending on the participants’ rating of the symptoms as not present (0), slight (1), mild (2), moderate (3), and severe (4)				
Craving tobacco smoking	156 (90.2)	17 (9.8)	2.618	0.106
Depressed mood	150 (92.6)	12 (7.4)	8.122	0.004 *
Frustration/anger	132 (92.3)	11 (7.7)	5.673	0.017 *
Fear and worry	130 (92.9)	10 (7.1)	6.763	0.009 *
Difficulty in focusing	144 (91.7)	13 (8.3)	5.246	0.022 *
Feeling nervous	128 (92.8)	10 (7.2)	6.339	0.012 *
Increased appetite	144 (92.3)	12 (7.7)	6.707	0.010 *
Difficulty sleeping	128 (90.8)	13 (9.2)	2.593	0.107
Impatience	127 (88.8)	16 (11.2)	0.441	0.507

^a^ Chi-square tests. * Significant at *p* < 0.05.

**Table 3:** Relationships between sociodemographic characteristics, vaping profile, daily amount of vaping, and habitual vaping behavior and level of motivation to quit vaping.

Variables	Motivation to Quit Vaping		OR ^a^	SE	95% CI	*p*-Value ^b^
	Low, *n* (%)	High, *n* (%)				
Monthly income, RM (USD)						
<RM 1000 (<225.30) (ref)	52 (19.1)	18 (46.2)	1.000			
RM 1000–1999 (225.30–450.38)	53 (19.5)	5 (12.8)	0.273	0.54	0.094–0.788	0.016 *
RM 2000–2999 (450.60–675.68)	73 (26.8)	6 (15.4)	0.237	0.51	0.088–0.639	0.004 *
RM 3000–3999 (675.90–900.98)	46 (16.9)	8 (20.5)	0.502	0.47	0.200–1.264	0.144
RM 4000–4999 (901.21–1126.28)	25 (9.2)	1 (2.6)	0.116	1.06	0.015–0.915	0.041 *
≥RM 5000 (1126.51)	23 (8.5)	1 (2.6)	0.126	1.06	0.016–0.998	0.050 *
Daily number of vaping sessions						
10 or less (ref)	97 (35.7)	22 (56.4)	1.000			
11–20	71 (26.1)	11 (28.2)	9.917	1.06	1.255–78.376	0.030 *
21–30	36 (13.2)	5 (12.8)	7.892	1.07	0.980–64.996	0.052
31 or above	68 (25.0)	1 (2.6)	10.928	1.12	1.211–98.612	0.033 *
Habitual vaping behavior						
Slight (ref)	32 (11.8)	14 (35.9)	1.000			
Mild	40 (14.7)	8 (20.5)	0.579	0.52	0.210–1.594	0.290
Moderate	109 (40.1)	11 (28.2)	0.279	0.48	0.110–0.708	0.007 *
Strong	66 (24.3)	4 (10.3)	0.185	0.64	0.052–0.654	0.009 *
Very strong	25 (9.2)	2 (5.1)	0.048	0.83	0.059–1.607	0.162
Perception of vaping being as satisfying as tobacco smoking						
No (ref)	117 (43.0)	26 (66.7)	1.000			
Yes	155 (57.0)	13 (33.3)	0.377	0.36	0.186–0.799	0.007 *

^a^ Unadjusted OR. ^b^ Logistic regression using the “Enter” method. * Significant at *p* < 0.05.

## Data Availability

Data can be requested from the corresponding author.
